# Basement Membrane-Rich Organoids with Functional Human Blood Vessels Are Permissive Niches for Human Breast Cancer Metastasis

**DOI:** 10.1371/journal.pone.0072957

**Published:** 2013-08-08

**Authors:** Rodrigo Fernández-Periáñez, Irene Molina-Privado, Federico Rojo, Irene Guijarro-Muñoz, Vanesa Alonso-Camino, Sandra Zazo, Marta Compte, Ana Álvarez-Cienfuegos, Ángel M. Cuesta, David Sánchez-Martín, Ana M. Álvarez-Méndez, Laura Sanz, Luis Álvarez-Vallina

**Affiliations:** 1 Molecular Immunology Unit, Hospital Universitario Puerta de Hierro, Majadahonda, Madrid, Spain; 2 Pathology Department, IIS-Fundación Jiménez Díaz, Madrid, Spain; 3 FEBIO Research Group, Universidad Complutense de Madrid, Madrid, Spain; University of Maastricht (UM), Netherlands

## Abstract

Metastasic breast cancer is the leading cause of death by malignancy in women worldwide. Tumor metastasis is a multistep process encompassing local invasion of cancer cells at primary tumor site, intravasation into the blood vessel, survival in systemic circulation, and extravasation across the endothelium to metastasize at a secondary site. However, only a small percentage of circulating cancer cells initiate metastatic colonies. This fact, together with the inaccessibility and structural complexity of target tissues has hampered the study of the later steps in cancer metastasis. In addition, most data are derived from *in vivo* models where critical steps such as intravasation/extravasation of human cancer cells are mediated by murine endothelial cells. Here, we developed a new mouse model to study the molecular and cellular mechanisms underlying late steps of the metastatic cascade. We have shown that a network of functional human blood vessels can be formed by co-implantation of human endothelial cells and mesenchymal cells, embedded within a reconstituted basement membrane-like matrix and inoculated subcutaneously into immunodeficient mice. The ability of circulating cancer cells to colonize these human vascularized organoids was next assessed in an orthotopic model of human breast cancer by bioluminescent imaging, molecular techniques and immunohistological analysis. We demonstrate that disseminated human breast cancer cells efficiently colonize organoids containing a functional microvessel network composed of human endothelial cells, connected to the mouse circulatory system. Human breast cancer cells could be clearly detected at different stages of the metastatic process: initial arrest in the human microvasculature, extravasation, and growth into avascular micrometastases. This new mouse model may help us to map the extravasation process with unprecedented detail, opening the way for the identification of relevant targets for therapeutic intervention.

## Introduction

Metastatic disease is the main cause of death in breast cancer patients [1]. Hematogenous cancer metastasis is a multistep process encompassing local invasion of cancer cells at primary tumors, intravasation into the blood vessel, survival in systemic circulation, extravasation, colonization and proliferation at the distant site, and ending with establishment of growing metastatic lesions [2]. The reduced number of cells involved at each step of the process along with the inaccessibility of the relevant anatomical sites has hampered the study of the mechanisms underlying metastasis. As breast cancer usually metastasizes to organs such as liver, lung, bone and brain, the ability to detect microfoci of cancer cells in an experimental setting is hindered by difficulty in imaging cells in these internal tissues [3]. In fact, most published studies have focused on the initial steps of metastasis (local invasion and intravasation) because they are more accessible for studying in currently existing animal models [4].

The use of luminescent and fluorescent proteins and developments in optical imaging technology are slowly facilitating the study of the sequential metastatic process [5]. Whole body bioluminescence imaging enables the dissemination of cancer cells through tissues to be monitored longitudinally in the same animal (but with low spatial resolution), whereas intravital microscopy (IVM), through confocal or multiphoton imaging of tumors expressing fluorescent proteins, enables spreading of a tumor to be observed with subcellular resolution. However, such IVM studies usually rely on dorsal skin chambers or reversible skins flaps to increase sensitivity of detection and are therefore limited to the primary tumor, providing only information over initial steps of metastasis. Obviously, this technique is not suitable for following tumor progression, and internal metastasis in a live intact animal due to the invasiveness of the procedure [5].

Consequently, the late steps in metastasis are poorly understood. Therefore, clarifying the molecular and cellular mechanisms underlying late steps of the metastatic cascade (i.e. post-arrest events) is a major focus in cancer research. In addition, all available experimental data are derived from *in vivo* models where critical steps in hematogenous metastasis of human cancer cells (intravasation and extravasation) are mediated by murine endothelial cells.

Recently, we and others have demonstrated that a network of functional human blood vessels can be formed in immunodeficient mice by co-implantation of primary human endothelial cells and human mesenchymal cells (MSC), embedded within a reconstituted basement membrane-like matrix [6-10]. Here, we demonstrate that BME-rich organoids containing functional microvessels composed of human endothelial cells support metastatic colonization in an orthotopic model of breast cancer. 

## Materials and Methods

### Ethics Statement

All experimental procedures were performed in accordance with the Spanish Government guidelines for the care and use of laboratory animals and were approved by the Hospital Universitario Puerta de Hierro Animal Care and Use Committee (CEBA).

### Cells

MDA-MB-231 (human breast adenocarcinoma; HTB-26) cells were obtained from the American Type Culture Collection (Rockville, MD, USA) and were cultured in Dulbecco’s modified Eagle’s medium (DMEM) supplemented with 10% heat inactivated FCS (all from Life Technologies, Gaithersburg, MD, USA). Human Umbilical Vein Endothelial Cells (HUVEC) were obtained from Lonza (Walkersville, MD, USA) and were cultured in EGM-2 medium containing 2% fetal bovine serum and endothelial cell growth supplements (Cambrex, Baltimore, MD, USA). Human bone marrow-derived MSCs were obtained from Inbiomed (San Sebastian, Spain) and cultured in low glucose DMEM supplemented with 10% FCS of selected lots.

### Lentiviral construction

An HIV-derived four-plasmid system was kindly provided by D. Trono (Department of Microbiology and Molecular Medicine, University of Geneva, Switzerland). The transfer vector pRRL-IRES-EGFP contains a cytomegalovirus (CMV) promoter that drives an enhanced-green fluorescent protein (EGFP) expression cassette [11]. The plasmid pRRL-F^Luc^-IRES-EGFP constructed as described previously [6], drives the expression of the firefly luciferase (F^Luc^) reporter gene. To construct the plasmid pRRL-R^Luc^-IRES-EGFP, the NheI/XbaI fragment derived from the plasmid pRL-CMV (Promega, Madison, WI, USA) was ligated into the XbaI digested backbone of plasmid pRRL-IRES-EGFP. The plasmid pRRL-R^Luc^-IRES-EGFP drives the expression of the renilla luciferase (R^Luc^) reporter gene. Lentiviral particles were produced by co-transfection of 293T cells through calcium phosphate precipitation, as described [12].

### Cell transduction

HUVEC passage 1 in normal growth media were seeded at a density of 7.5 × 10^3^ cells/cm^2^, allowed to adhere and infected overnight with lentivirus (F^Luc^-IRES-EGFP) at a final multiplicity of infection (MOI) of 10. HUVEC^Fluc^ were used *in vivo* in passages 3-5. MDA-MB-231 cells in normal growth media were seeded at a density of 6.25 × 10^4^ cells/cm^2^, allowed to adhere and infected overnight with the lentivirus R^Luc^-IRES-EGFP at a final MOI of 10.

### Cell culture assays for luciferase activity

For bioluminescence imaging (BLI) of live intact cells (HUVEC^Fluc^ or MDA-MB-231^Rluc^), cell suspensions were serially diluted into appropriate cell culture media in 96-well plates (ranging from 6400 to 100 cells/well) and analyzed in triplicate. After the addition of 20 µg/well of the appropriate substrate (D-Luciferin or coelenterazine; Promega), bioluminescence was quantified using an Infinite 200 microplate luminometer (Tecan, Switzerland).

### Endothelial cell proliferation assay

Endothelial cell proliferation assay was performed by seeding HUVEC or HUVEC^Fluc^ (500 cells/well) in 96-well plates in complete HUVEC medium. After 24 hours the cells were switched to fresh EBM-2 basal medium with 0.1% FCS and incubated for 24 hours, then the cells were switched to fresh EBM-2 with 0.5% FCS in the absence or presence of 20 ng/mL of VEGF (Peprotech, London, UK). The cells were cultured for an additional 48 hours, and viable cells were detected using the MTT colorimetric assay (Sigma-Aldrich, St. Louis, MO, USA).

### Endothelial cell tube formation assay

Basement Membrane Extract (BME) (Matrigel, BD Biosciences) was plated at 4 ^°^C in 96-well plates, and allowed to gellify for 30 min at 37 ^°^C, then HUVEC or HUVEC^Fluc^ in 1% EGM-2 medium, were plated (1.5 x 10^4^ cells/well) and cultured for 14-16 hours. Formation of capillary-like structures was assessed by bright-field and fluorescence microscopy.

### Flow cytometry

For phenotypic analysis cells were treated with appropriate dilutions of fluorochrome-conjugated mAbs (Table S1) for 30 min at 4 ^°^C. All samples were fixed in 2% formaldehyde and analyzed with a Beckman-Coulter FC-500 Analyzer (Beckman Coulter, Fullerton, California, USA).

### Animal studies

Six-week-old female athymic nude mice (Hsd: athymic Nude/Nude; Harlan Ibérica, Barcelona, Spain) were used for abdominal wall implantation and for xenograft experiments. Mice were kept under anesthesia during all manipulations and all efforts were made to minimize suffering. Anesthesia was induced in an induction chamber with 2.5% isofluorane in 100% oxygen at a flow rate of 1 L/min and maintained with a 1.5% mixture at 0.5 L/min. Animals were housed under pathogen-free conditions and were given irradiated food and autoclaved water *ad libitum*. For abdominal implantation of human BME-rich vascularized organoid (HVO) a mixture (ratio 4:1) of HUVEC^Fluc^ (3 x 10^5^) and MSC (7.5 x 10^4^), were suspended in 150 µl BME (Matrigel) and inoculated subcutaneously in the ventral area (right lower quadrant). For abdominal implantation of BME-rich control organoids (CO) 150 µl of Matrigel were inoculated subcutaneously in the ventral area (left lower quadrant). All batches of Matrigel were adjusted to 8 mg/ml by addition of PBS and supplemented with heparin (128 U/ml) (Sigma-Aldrich), human VEGF (50 ng/ml) and human bFGF (150 ng/ml) (both from PeproTech). MDA-MB231^Rluc^ cells (2 x 10^6^) were injected directly into the second left mammary fat pad of each mouse, 48 hours after abdominal wall HVO implantation. Tumor growth and metastasis spread was monitored every week by R^Luc^ BLI for up to 10 weeks. Tumors were measured twice weekly using precision calipers. Tumor volume was calculated using the formula: width^2^ x length x 0.52. The functionality of HVO was monitored every two weeks by F^Luc^ BLI up to 10 weeks.

### In vivo and ex vivo bioluminescence imaging

Mice were imaged using the high-resolution charge-coupled-device (CCD) cooled digital camera ORCA-2BT (Hamamatsu Photonics France, Massy, France), and Hokawo software (Hamamatsu Photonics). The mice were anesthetized using inhaled isoflurane, as described above, injected i.v. with 125 mg/kg (100 µl) D-luciferin dissolved in PBS or with 900 mg/kg (100 µl) coelenterazine-based compound dissolved in PBS, and placed on a thermostated bed. BLI was collected with 1 min integration time in F^Luc^ imaging and with 5 min integration time in R^Luc^ imaging and pseudocolor representations of light intensity were superimposed over the grayscale reference image acquired at low light (exposure time 20 ms). An average of 6 kinetic BLI acquisitions was collected after substrate injection to confirm a peak of photon emission. For quantitation of the detected light, regions of interest were drawn and the light emitted from each region was recorded by recording the total number of photons per second (total flux) after background subtraction. At the end of each experiment anesthetized animals were sacrificed and BME-rich organoids (CO and HVO), inguinal lymph node and the different organs (lung, liver and spleen) were rapidly harvested and processed. Organs and BME-rich organoids were placed in PBS containing coelenterazine-based compound and individually and immediately scanned for the presence of R^Luc^ bioluminescent metastatic cells during 1 min of integration time as described above.

### RNA isolation and real-time quantitative PCR

Total RNA isolation of tumor and organs was performed using the *RNeasy Plus Mini Kit* (Qiagen GmbH, Hilden, Germany) or the *RNeasy Plus Micro Kit* (Qiagen) according to the weight of the sample used for the RNA extraction. For disruption and homogenization of samples, *MagNA Lyser Green Beads* (Roche Diagnostics, IN, USA) were used according to the manufacturer’s protocol. cDNA was synthesized using 0.1 mg of total RNA by random primed reverse transcription with *SuperScript VILO cDNA Synthesis kit* for RT–PCR (Roche Applied Science, Germany) according to the recommended protocol. Real-time quantitative PCR (RT-qPCR) was performed in a LightCycler 480 apparatus (Roche Applied Science) using the *LightCycler 480 SYBR Green I Master kit* (Roche Applied Science). mRNA expression in each sample was measured as a ratio against the geometric average of the reference housekeeping human gene succinate dehydrogenase complex subunit A (SDHA). The relative concentrations of the target and the reference genes were calculated by interpolation using a standard curve of each gene plotted from the same serial dilution of cDNA. PCR primers (Table S2) were designed with Primer Express software (Applied Biosystems, Foster City, CA, USA) or Universal ProbeLibrary Assay Design Center (Roche Applied Science). The resulting amplicons had a size of approximately 100 pb.

### Immunohistochemistry

Immunohistochemical analyses were performed on 5 µm formalin-fixed, paraffin-embedded tissue sections. Briefly, samples were deparaffinized, re-hydrated and subjected to antigen retrieval procedures as described previously [12]. A histological section from each initial tissue specimen was stained with hematoxylin and eosin according to standard protocols. The mAbs used are listed in Table S3. Primary mouse mAbs were detected with the *Mouse on Mouse* (*M.O.M.*) *peroxidase kit* (Vector Laboratories, Burlingame, CA, USA). Primary rat mAbs were detected with the *Vectastain® ABC kit* for rat IgG (Vector Laboratories). As a chromogenic substrate, DAB (Vector Laboratories) was used, followed by hematoxylin counterstaining. Staining was evaluated using Leica DM E microscope (Leica Microsystems, Jena, Germany). Images were acquired using Leica ICC50 HD camera and processed by Leica LAS EZ software. The number of CD45^+^ cells was evaluated quantitatively in four randomly chosen areas of BME-rich organoids (CO and HVO).

### Quantitative analyses of vascular morphogenesis

Vascular structures in BME-rich organoids (HVO and CO) were classified as either immature or mature vessels. Mature vessels were defined as vessel structures containing erythrocytes. Vascular morphogenesis was quantified by enumerating the mature vessels in four randomly chosen fields and expressing the mean ± SD obtained for each condition. In order to assess vascular leakiness, extravascular red blood cells (RBC) were quantified in four randomly chosen fields and mean ± SD obtained for each organoid type is shown. Extravascular RBC are those erythrocytes localized inside the organoids but unrelated to vascular structures.

### Immunofluorescence

Immunofluorescence analysis was performed on formalin-fixed paraffin-embedded 3 µm tissue sections. After deparaffinization, heat antigen retrieval was performed in pH 9.0 EDTA-based buffer. Human CD44 was detected using the rabbit mAb clone EPR1013Y (Table S3) and an Alexa Fluor 655 conjugated goat anti-rabbit IgG antibody (Molecular Probes, Life Technologies). Human CD34 was detected using the mouse mAb clone QBEnd10 (Table S3), and an Alexa fluor 488 conjugated goat anti-mouse IgG antibody. Sections were counterstained with DAPI (Abbott Molecular, Abbott, Park, IL, USA) to visualize cell nuclei. Fluorescence assays were performed at room temperature using a Dako Autostainer (Dako, Carpinteria, CA, USA). Staining was evaluated by two investigators (F.R. and S.Z.) using a Leica fluorescence DM2000 microscope and images were obtained and processed by Nuance FX Multispectral Imaging System (CRi, Caliper, Hopkinton, MA, USA).

### Statistics

The mean and standard deviation (SD) were calculated by using Prism V (Graphpad Software, San Diego, CA, USA), and error bars represent the SD. Each experiment was performed a minimum of three times. The data were evaluated by t test and were considered to be statistically significant when p <0.05.

## Results

### Establishment and histological analysis of human vascularized organoids

**Figure 1:**
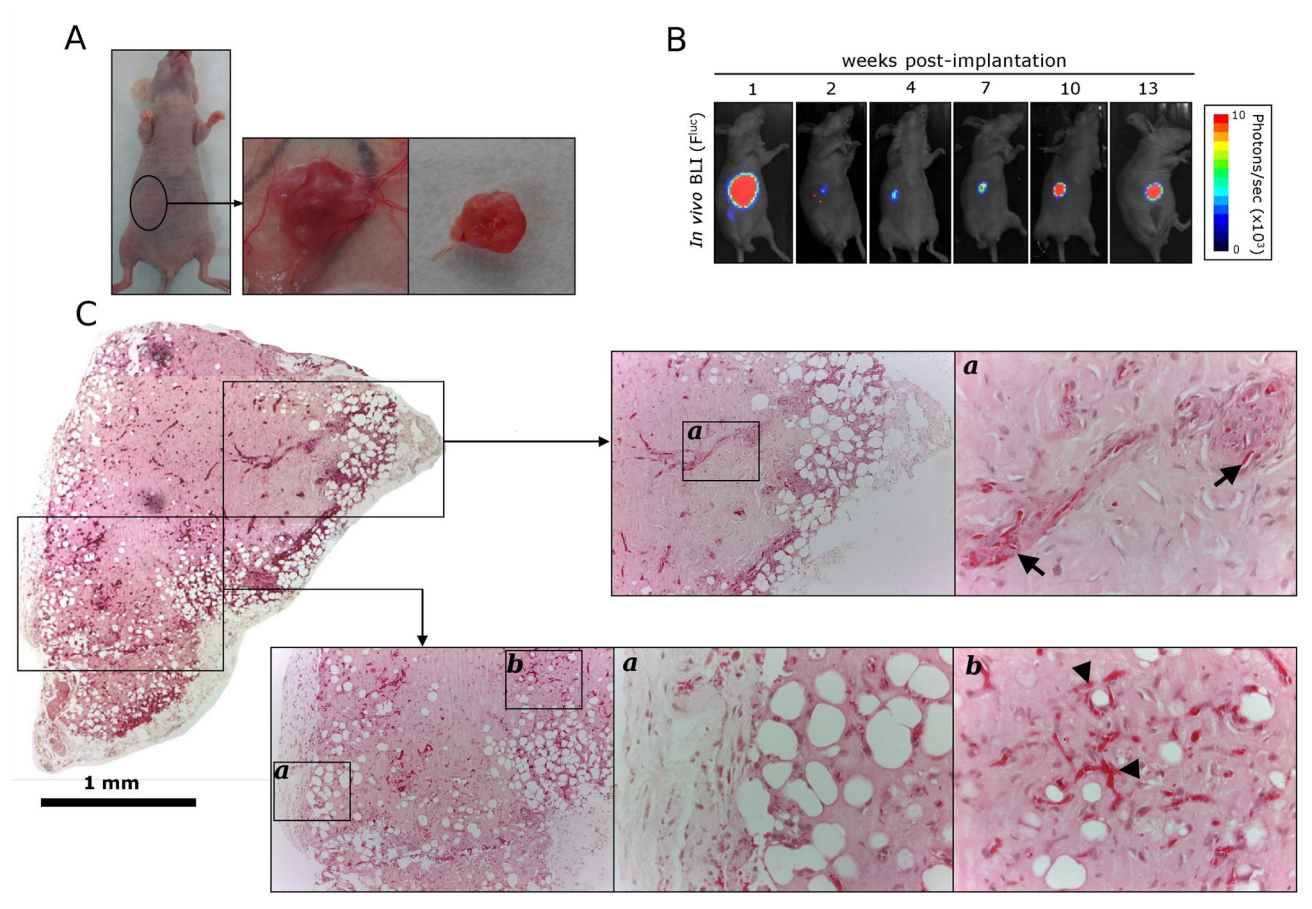
Generation of BME-rich organoids with functional human blood vessels in immunodeficient mice. (a) A mixture of HUVEC^FLuc^ (3 x 10^5^) and human MSC (7.5 x 10^4^) were embedded in supplemented BME (150 µl) and inoculated subcutaneously in the ventral area (right lower quadrant) of immunodeficient mice. Human vascularized BME-rich organoids were easily identified as small bumps under the skin at all times *in vivo*. (b) Ventral D-luciferin-based F^Luc^-BLI images of a representative mouse 1, 2, 4, 7, 10 and 13 weeks after implantation of the human vascularized BME-rich organoid. (c) Low magnification (4x) micrograph displaying the entire organoid (Scale bar is 1 mm). Hematoxylin and eosin (H and E)-stained sections taken from different part of the organoids revealed the presence of numerous luminal structures containing erythrocytes (arrowheads), and some glomeruloid microvascular proliferations (arrows). 10x and 40x images are shown.

Confirming our previous work [6,10] we found that the co-implantation of genetically modified human umbilical vein endothelial cells (HUVEC) harboring the firefly luciferase (F^Luc^) gene (HUVEC^FLuc^) and human bone marrow derived MSC (Figure S1-S4) embedded within a reconstituted basement membrane extract (BME, also known as Matrigel) supplemented with VEGF, bFGF and heparin and inoculated subcutaneously into nude mice (Figure 1a), results in the formation of mature, stable human blood vessels. These vessels are functional, meaning that they are able to anastomose with preexisting mouse vessels, and can be assessed non-invasively and quantitatively by *in vivo* bioluminescent imaging (BLI) (Figure 1b). The temporal silencing of luciferase activity and subsequent recovery is related to the lapse of time required for the establishment of an incipient vascular network and its integration into the mouse vascular bed [6].

**Figure 2:**
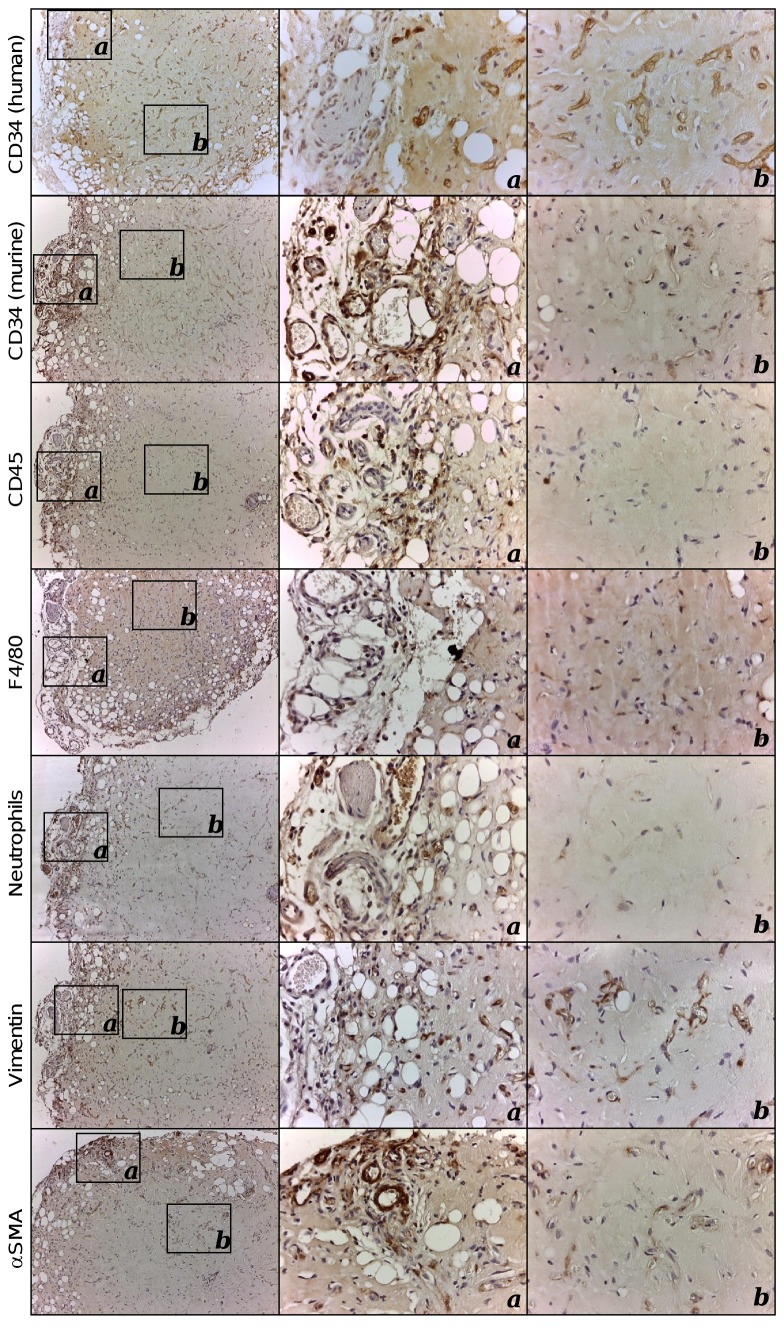
Immunohistochemical characterization of human vascularized BME-rich organoids. Immunohistochemical characterization of explanted human vascularized BME-rich organoids using anti-CD34 (species specific: human and mouse; [39]), anti-CD45, anti-F4/80, anti-neutrophils, anti-vimentin and anti-α-SMA antibodies (Table S3). Cell nuclei were counterstained with hematoxylin. 10x and 40x images are shown.

In this paper we performed a detailed histological characterization of the implants. The injection of 150 µl of supplemented BME containing HUVEC^FLuc^ and MSC, originated the formation of very stable organoids (Figure 1a), with volumes ranging from 13.7 to 25.5 mm^3^. The mean was 19.8 ± 5.1 (SD). Two main areas can be distinguished: a central homogeneous region, with an extensive network of perfused blood vessels (containing erythrocytes; Figure 1c arrowheads) and few non-vascular cells, and a peripheral region with appreciable adipogenesis [13], in close contact with the subcutaneous tissue of the host (Figure 1c). The network of capillary-like microvessels with a clear endothelial lining that expressed human CD34, was distributed rather uniformly throughout the organoid, while murine vessels were distributed almost exclusively in the periphery of the organoid and the surrounding host tissues (Figure 2). Interestingly, some human blood vessels constituted poorly organized vascular structures that mimic renal glomeruli (Figure 1c, arrow). These structures have been named as “glomeruloid microvascular proliferations”, and represent a type of angiogenic blood vessel [14]. The human vascularized BME-rich organoids (HVO) were well tolerated by the host and did not evoke adverse inflammatory responses. A weak inflammatory infiltrate (Figure S5) constituted mainly by macrophages and neutrophils was present in the periphery of the organoid (Figure 2), whereas B lymphocytes, NK cells and cells from other hematopoietic lineages were not present (data not shown). Finally, interstitial cells showed a myofibroblast phenotype [vimentin and α-smooth muscle actin (SMA)], and were interspersed in the organoid (Figure 2). Furthermore, with the exception of an increase in vascular density, and adipocyte size and number (Figure S6), the general structure of the organoid and the amount of infiltrating cells were unchanged for more than 60 days (Figure S7).

**Figure 3:**
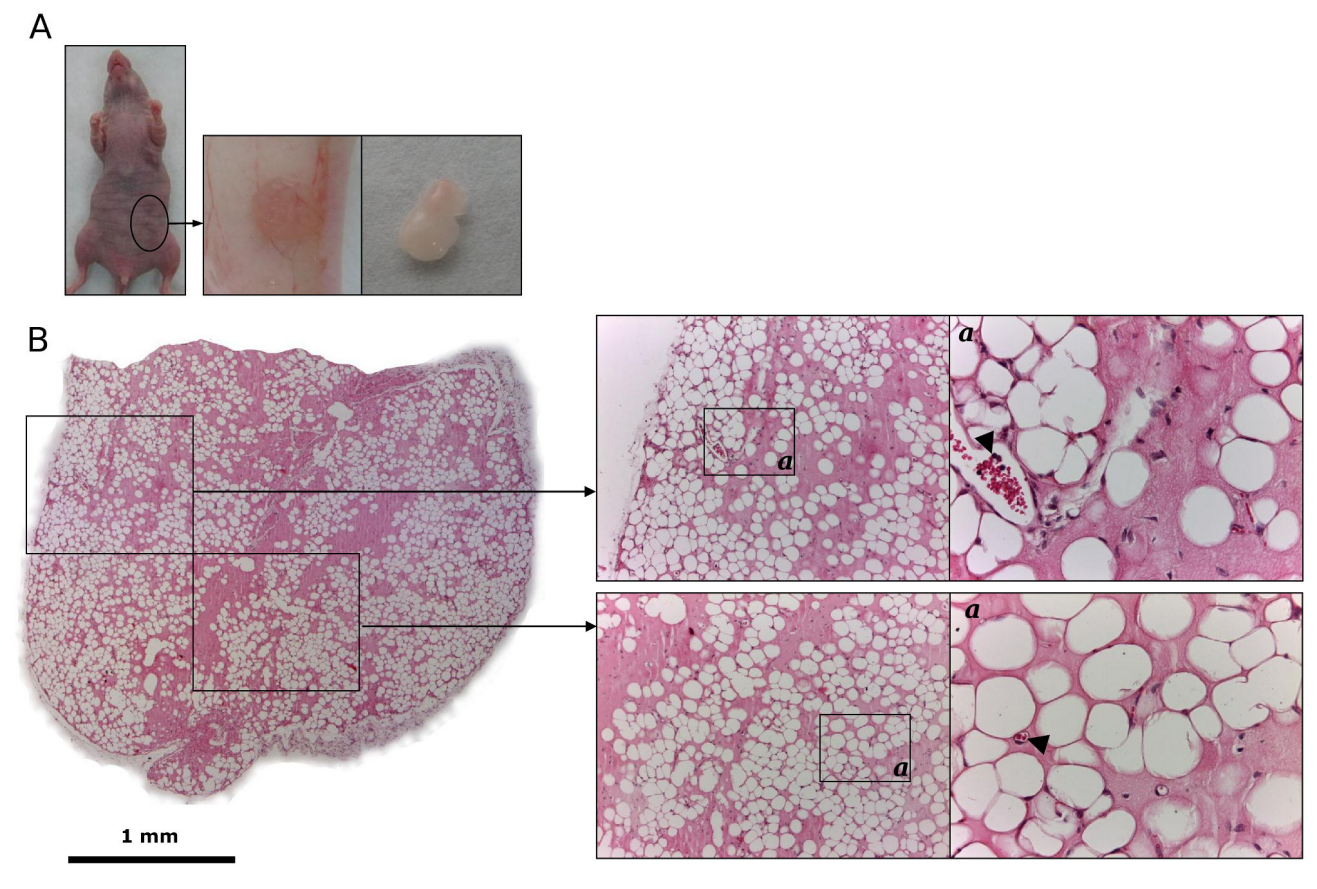
Generation of control BME-rich organoids without human cells in immunodeficient mice. (a) Supplemented BME (150 μl) was inoculated subcutaneously in the ventral area (left lower quadrant) of immunodeficient mice. Control BME-rich organoids were easily identified as small bumps under the skin at all times *in vivo*. (b) Low magnification (4x) micrograph displaying the entire organoid (Scale bar is 1 mm). Hematoxylin and eosin (H and E)-stained sections taken from different part of the control organoids revealed the presence of numerous adipocytes, and some luminal structures containing erythrocytes (arrowheads). 10x and 40x images are shown.

**Figure 4:**
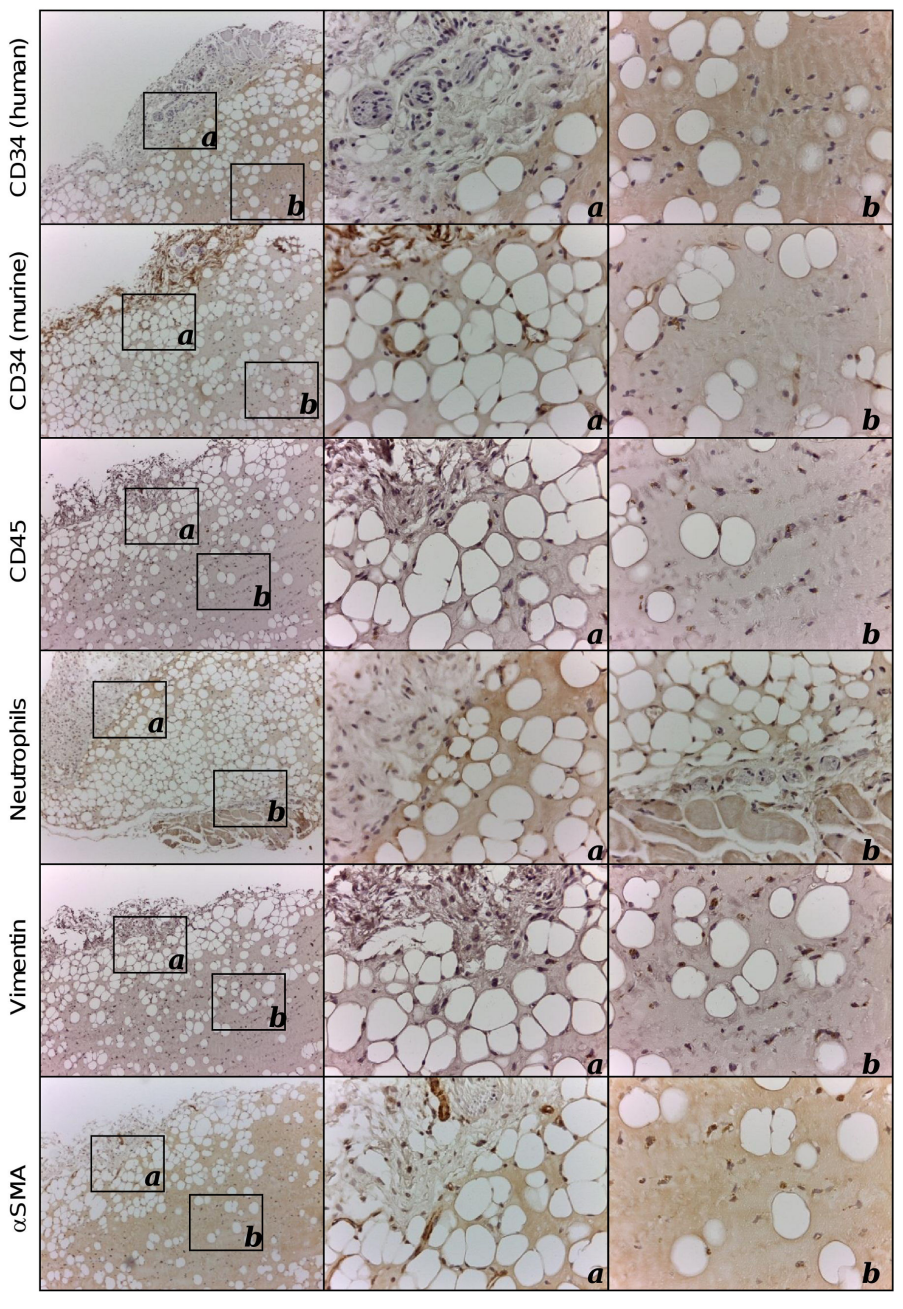
Immunohistochemical characterization of control BME-rich organoids. Immunohistochemical characterization of explanted control BME-rich organoids using anti-CD34 (species specific: human and mouse; [39]), anti-CD45, anti-neutrophils, anti-vimentin and anti-α-SMA antibodies (Table S3). Cell nuclei were counterstained with hematoxylin. 10x and 40x images are shown.

As shown in Figure 3, the subcutaneous implantation of 150 µl of supplemented BME alone resulted in the formation of organoids with a volume similar to HVO (average volume 17.6 ± 1.6 mm^3^). However, control organoids (CO) without human cells (HUVEC^FLuc^ and MSC) were pale, indicating no or little blood vessel formation (Figure 3a). Histological analysis confirmed that the CO were colonized by murine cells but the degree of vascularization (neo-vessel density and complexity) was significantly lower than that observed in HVO (p < 0.05, Figure S8a). By contrast vascular leakiness, quantitatively assessed by counting the number of extravascular erythrocytes, was significantly higher in CO (p < 0.05, Figure S8b and c) than in HVO. A marked adipogenesis was observed in all areas of the CO sections (Figure 3b). Immunohistochemical staining demonstrated that the peripheral region exhibited a dense CD34 positive vascular network that contains luminal vessels filled with erytrocytes (Figure 3b arrowheads), and a weak inflammatory infiltrate (Figure 4) similar to that observed in HVO (Figure S5).

### Human vascularized organoids are colonized more efficiently by disseminated human breast cancer cells than control organoids

**Figure 5:**
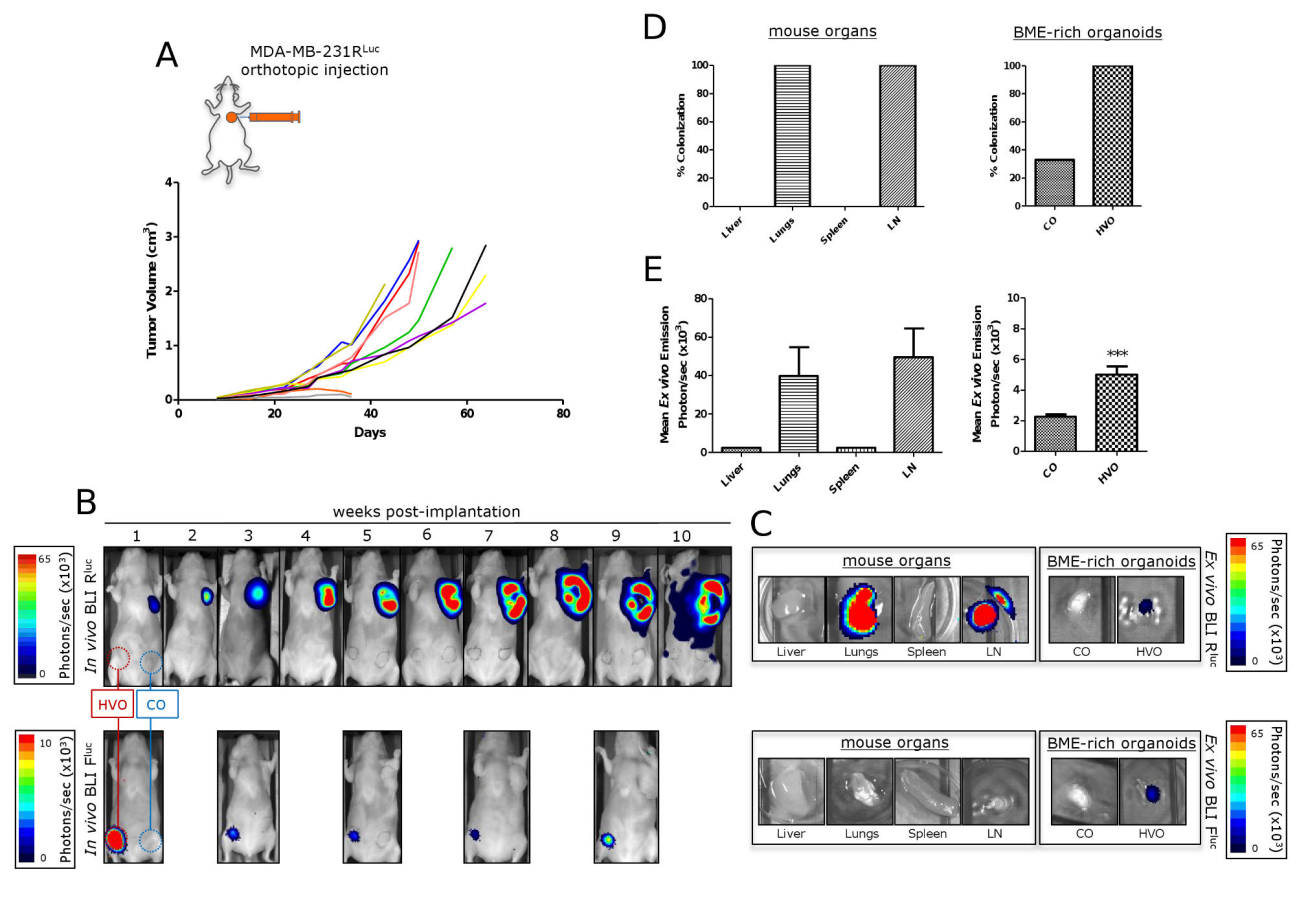
Spontaneous metastasis from primary mammary fat pad tumors. MDA-MB-231^RLuc^ human breast cancer cells were implanted into the orthotopic environment of the left axillary MFP of nude mice bearing human vascularized BME-rich organoids (HVO) and control BME-rich organoids (CO), without human cells. Primary tumor growth (a, b), metastatic spread (b-e) and HVO functionality (b, c) were monitored over time *in vivo* and *ex vivo*. (a) Tumor growth curves for MDA-MB-231^RLuc^ (*n = 10*) orthotopic mammary tumors. (b) *In vivo* ventral coelenterazine-based R^Luc^-BLI images (upper panels) and D-luciferin-based F^Luc^-BLI images (lower panels) of a representative mouse. (c) *Ex vivo* coelenterazine-based R^Luc^-BLI images (upper panels) and D-luciferin-based F^Luc^-BLI images (lower panels) of excised lungs, inguinal lymph node (LN), spleen, liver, HVO and CO of a representative mouse. (d) Percentage of metastatic colonization and (e) tumor burden in harvested tissues (normal mouse organs and BME-rich organoids) of mice (*n = 8*) with primary tumor growth. Significant differences (*** p < 0.001).

The ability of circulating cancer cells to colonize HVO and CO was next assessed in an orthotopic model of human breast cancer. Implantation of MDA-MB-231 human breast cancer cells into the second left mammary fat pad (MFP) of 6-8 weeks old female athymic nude mice is a well-characterized spontaneous metastasis model [15,16]. The take-rate percentage of genetically modified MDA-MB-231 cells expressing the renilla luciferase (R^Luc^) gene (MDA-MB-231^RLuc^) (Figure S1-S2) in the MFP orthotopic xenograft model was 80% (*n = 10*), and the growth kinetics was examined over the subsequent 6–10 weeks (Figure 5a). Mice were evaluated for primary tumor growth (caliper method and *in vivo* BLI), HVO functionality and spontaneous metastasis (*in vivo* and *ex vivo* BLI).

MDA-MB-231^RLuc^ mammary tumor bioluminescence started to increase about 3 weeks after tumor cell injection (Figure 5b, upper panels). Metastatic signals also appeared at that time in the left axillary region in 33% of the mice, indicating spontaneous ipsilateral thoracic metastasis. Subsequent thoracic images demonstrated a progressive increase in metastatic spread over time, and by week 6 half of the animals had developed bilateral thoracic metastases, visible by *in vivo* BLI (Figure 5b). By 7–10 weeks, metastatic signals appeared in the inguinal region in 50% of the mice. HVO remained functional throughout the study period (Figure 5b, lower panels).

Representative *ex vivo* images are shown in Figure 5c. As shown in Figure 5d metastases were detected in lungs, inguinal lymph nodes and HVO in all mice (*n = 8*). By contrast only a third of CO were colonized, while no metastases were detected in the liver or spleen. Figure 5e shows quantification of light emission by excised tissues. The metastatic burden was significantly higher in inguinal lymph node and lungs that in HVO. However, HVO *ex vivo* signals were significantly higher than those observed in the CO (p < 0.001). Subsequent histological evaluation confirmed extensive metastases through the lung tissue. To determine which antibody was best suited to define the metastatic deposits, we performed immunohistochemical studies with anti-CD44 and anti-R^Luc^ antibodies. MDA-MB-231^RLuc^ cells expressed high levels of CD44 respectively on the cell surface (Figure S9a), whereas R^Luc^ was found in the cytoplasm (Figure S9b). Our results indicate that the anti-CD44 mAb provides the best definition of the metastatic breast cancer cells, and allows the identification of metastases of varying sizes (Figure S9c).

### Molecular detection and characterization of disseminated tumor cells and micrometastases in human vascularized organoids

**Figure 6:**
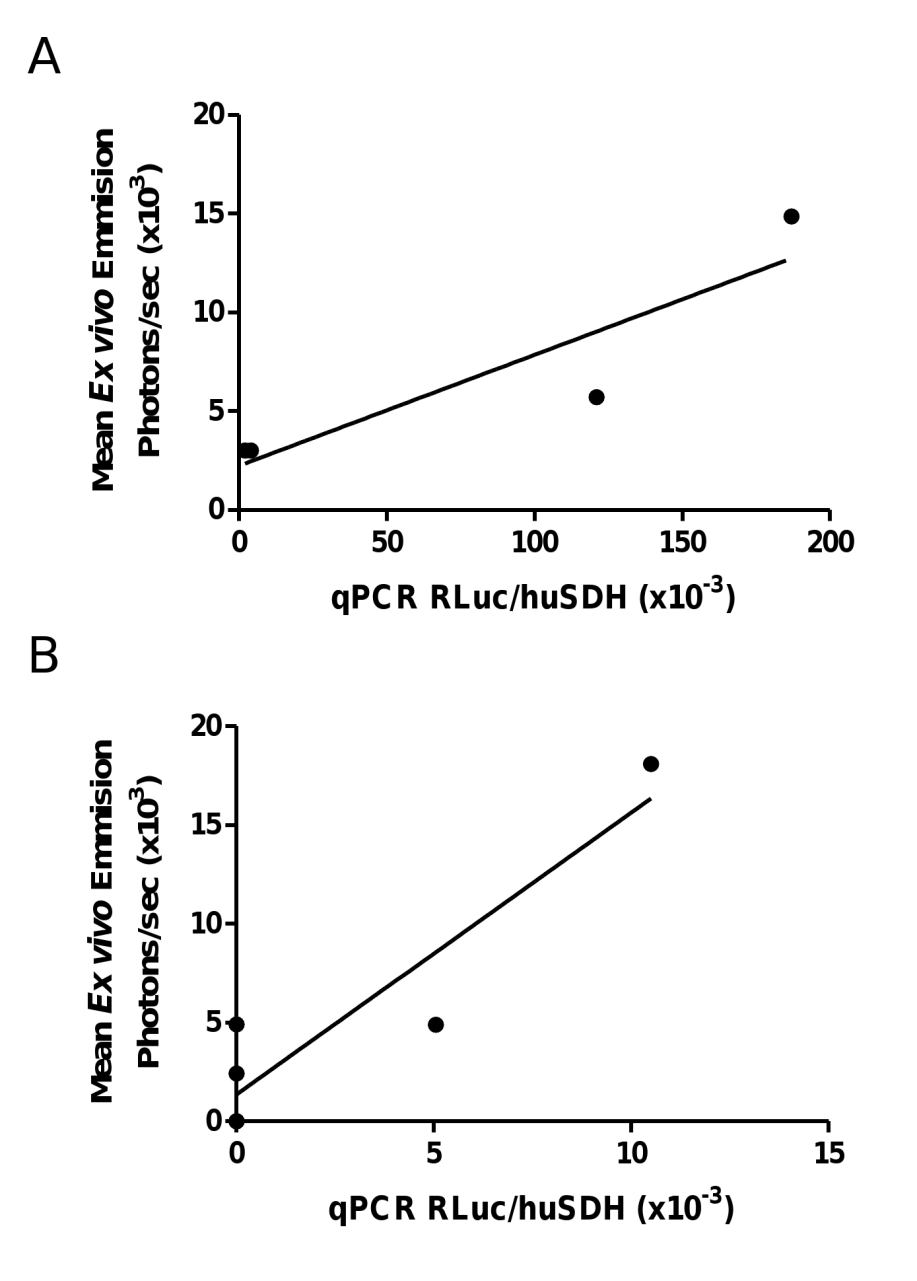
Correlation between mRNA levels of Renilla Luciferase (R^Luc^) and *ex vivo* coelenterazine-based R^Luc^-bioluminescence of lungs (*n = 4*) and human vascularized BME-rich organoids (*n = 5*). In both cases R^Luc^ mRNA concentrations were well correlated (a: r = 0.829; b: r = 0.851) with *ex vivo* bioluminescence data.

The presence of MDA-MB-231^RLuc^ cells in the lungs and HVOs was assessed as a function of R^Luc^ expression relative to human succinate dehydrogenase A (SDHA). R^Luc^ and SDHA mRNA levels were quantified by quantitative reverse transcription-PCR. In both cases R^Luc^ mRNA concentrations were well correlated (r = 0.82 and 0.85, respectively) with *ex vivo* bioluminescence (Figure 6).

**Figure 7:**
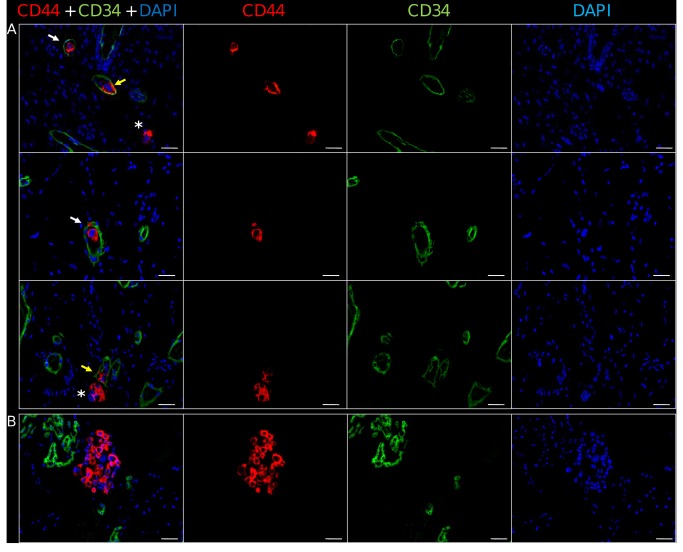
Detection of disseminated tumor cells in human vascularized BME-rich organoids **sections**. (a) The parenchyma contains a network of human CD34^+^ capillary-like microvessels (in green) and numerous isolated or clusters of CD44^+^ MDA-MB-231 tumor cells (in red), which are circulating (white arrow), attached to the endothelium (yellow arrow) or extravasating and colonizing the surrounding matrix (asterisk). (b) Small foci of CD44^+^ MDA-MB-231 tumor cells adjacent to human CD34^+^ capillary-like microvessels and glomeruloid microvascular proliferations. Nuclei are labeled in DAPI, x200. Scale bar is 25 μm.

To further confirm the presence of MDA-MB-231^RLuc^ cells in the HVOs, and to study their distribution, we performed immunofluorescence studies in those HVO with the highest *ex vivo* bioluminescence emission (*n = 3*). As shown in Figure 7a, randomly distributed isolated or small clusters (mean = 3.1 ± 1.7 SD) of tumor cells were detected through the HVO inside the human CD34^+^ microvessels. These intravascular CD44^+^ cells were either circulating, attached to the endothelium or trapped in the capillary-like microvessels. Notably some of the intravascular MDA-MB-231^RLuc^ cells traversed the human vascular wall to colonize the surrounding matrix (Figure 7a). Moreover, in addition to these micrometastases we identified larger growing metastases, composed by 40-50 CD44^+^ tumor cells adjacent to blood vessels of human origin (Figure 7b).

## Discussion

We have herein described a new mouse model to study metastatic colonization, and we demonstrate that disseminated human breast cancer cells efficiently colonize BME-rich organoids containing a functional microvessel network composed of human endothelial cells connected to the mouse circulatory system. Despite their apparent structural simplicity, HVO were efficiently colonized by MDA-MB-231 cells, and importantly, human breast cancer cells could be unequivocally detected at different stages of the metastatic process: initial cell arrest in human capillaries, extravasation, and growth from avascular micrometastases to vascularized micrometastases growing into the surrounding BME. In control BME-rich organoids without human cells, the number of vessels was lower than in the HVO and these were more immature and leaky. Despite increased leakiness of vessels in CO, the percentage of metastatic colonization was significantly lower than that observed in HVO, although we cannot rule out that differences in microvascular density may contribute to differences in the number of metastasis.

Tumor-derived BME has been used for more than 20 years in models (both *in vitro* and *in vivo*) of angiogenesis, invasion and primary tumor growth [15-17]. Proteomics analysis of tumor-derived BME resulted in the identification of 1302 unique proteins [20], but in fact it is composed mainly of laminin-111 (56%), collagen IV (31%), entactin (8%) and perlecan (4%) [21]. These components constitute what has been called the “basement membrane toolkit” [22], which is found in most metazoans and has been shown to be crucial to their evolution. Given that the basement membrane is the first extracellular matrix (ECM) produced in the developing embryo, it was quickly identified as an important factor for modulating stem cell behavior [23], that plays multiple roles in the stem cell niche. It is perhaps not surprising then that BME is the gold standard for the maintenance of a wide array of human embryonic stem cell (hESC) lines, as it contains all of the proteins that have been shown to promote hESC growth when used individually [24].

Furthermore, increasing evidence suggests that the ECM is also a key component of the cancer stem cell niche [25]. In fact, the ECM not only promotes cancer cell growth at the primary site but also provide signals that support metastatic cell survival, colonization and proliferation [25]. Several reports have recently revealed that the ECM proteins fibronectin, tenascin-C, periostin and versican play essential roles as components of the metastatic niche for tumor-initiating cells that invade the lungs [25–29]. Not surprisingly, BME has been widely used to test the metastatic potential of cancer cells *in vitro* [18].

During the past several years, a crucial role for bone-marrow derived cells in priming distant tissues for tumor engraftment has been uncovered [26]. VEGFR1^+^ hematopoietic progenitor cells are mobilized and recruited early during the formation of pre-metastatic niche [26]. A causal relationship between chronic inflammation and metastasis formation has been proposed [30], and accordingly a variety of inflammatory cells (CD11b^+^ myelod cells, monocytes/macrophages, lymphocytes and neutrophils) that drive primary tumor growth may have an important role in metastatic colonization [31,32]. In addition stromal cells [33,34] and endothelial cells [35,36] also participate in the metastatic niche. Although we have not studied the contribution of host-derived cells in the colonization of HVO, this model could be an ideal system for studying the role of different cell types in the process of metastatic colonization.

Molecular interactions between human breast cancer cells and murine endothelial cells may not recapitulate those taking place during intravasation and extravasation of cancer cells in patients. For this reason, we estimate that mice carrying human vascularized BME-rich organoids would be a more suitable model for studying the interactions between disseminated cancer cells and host endothelium. In fact, a murine microenvironment is not always a “congenial soil” for metastatic human cells [37]. For example, in a mouse model of human breast cancer, bone metastases were more frequent when the target organ was of human origin, suggesting a species-specific tropism [38].

In summary, we have described here the generation and characterization of a simple, non-invasive mouse model that could more accurately recapitulate human breast cancer metastasis, and could be easily adapted for intravital microscopy. Although this model is still artificial, it may help to assess the cancer cell extravasation step in a more physiological setting than previous models, opening new avenues to the development of innovative anti-cancer strategies.

## Supporting Information

Figure S1 **Schematic representation of lentiviral bicistronic vectors.** Schematic representation of the lentiviral bicistronic vectors pRRL-F^Luc^-IRES-EGFP containing firefly luciferase (F^Luc^) and enhanced-green fluorescent protein (EGFP) genes (a), and pRRL-R^Luc^-IRES-EGFP containing renilla luciferase (R^Luc^) and EGFP genes (b). LTR, long terminal repeat; ΔGAG, ATG-deleted group-specific antigen; RRE, Rev-responsive element; EMCV IRES, encephalomyocarditis virus internal ribosomal entry site.
(TIF)

Figure S2 **EGFP expression and bioluminescent properties of HUVEC^FLuc^ and MDA-MB-231^RLuc^ cells.** Flow cytometry analysis showing EGFP expression by lentivirally transduced HUVEC^FLuc^ (a) or MDA-MB-231^RLuc^ (c) cells. Bioluminescent properties of HUVEC^FLuc^ (b) or MDA-MB-231^RLuc^ cells (d) in the presence and absence of substrate (D-luciferin or coelenterazine). Luciferase activity is expressed as relative light units (RLU). Data represent the average ± SD of triplicate samples.
(TIF)

Figure S3 **Comparative study of wild-type HUVEC and lentivirally transduced HUVEC^FLuc^.** Flow cytometry alaysis of CD34 or CD31 expression (Table S1) on HUVEC (a) and HUVEC^FLuc^ (b). Isotype-matched antibodies were used as control (grey line). VEGF significantly induced HUVEC (c) and HUVEC^FLuc^ (d) proliferation. Data represent the mean ± SD of triplicate samples. The differences were statistically significant (*p < 0.05, **p < 0.01). Formation of capillary-like structures by HUVEC (e) and HUVEC^FLuc^ (f) cultured for 14-16 h on reconstituted Matrigel.
(TIF)

Figure S4 **Phenotype of bone marrow-derived human MSC.** Cells were trypsinized, labeled with antibodies against the indicated antigens (Table S1) and analyzed by flow cytometry. Isotype-matched antibodies were used as control (grey line).
(TIF)

Figure S5 **Comparative analysis of CD45^+^ cells in human vascularized and control BME-rich organoids.** Mean ± SD of cells stained with anti-CD45 antibody (Table S3) in four randomly chosen fields (*n* = 3).
(TIF)

Figure S6 **Temporal changes in mature vessel density and in the total adipocyte number in human vascularized BME-rich organoids.** Temporal changes in mature vessel density (mean ± SD of perfused vessels in four randomly chosen fields, *n* = 3) (a), and the number of adipocytes per square millimeter (mean ± SD, *n* = 3) (b).
(TIF)

Figure S7 **Hematoxylin and eosin-stained sections of human vascularized BME-rich organoids.** 15 days (a), 30 days (b), and 45 days after implantation. 4x and 10x images are shown.
(TIF)

Figure S8 **Comparative analysis of mature vessel density and vascular leakiness in human vascularized and control BME-rich organoids 30 days after implantation.** (a) Mean ± SD of perfused vessels in four randomly chosen fields (*n* = 3). (b) Mean ± SD of extravascular red blood cells (RBC) in four randomly chosen fields (*n* = 3). Significant differences (* p < 0.05). (c) Hematoxylin and eosin-stained sections and immunohystochemical characterization of explanted BME-rich human vascularized and control organoids using anti-CD34 (species specific: human and mouse [39]).
(TIF)

Figure S9 **Characterization of antibodies against CD44 and Renilla Luciferase (R^Luc^).** (a) Cell surface expression of CD44 (MEM-85) in MDA-MB-231^RLuc^ cells. (b-c) Immunohistochemical staining of lung metastases. Serial sections of lung tissue were stained for CD44 (F10-44-2) and R^Luc^. 10x and 40x images are shown.
(TIF)

Table S1 **Fluorochrome-conjugated monoclonal antibodies.**
(DOCX)

Table S2 **Primer pairs used for real-time quantitative RT-PCR.**
(DOCX)

Table S3 **Unconjugated monoclonal antibodies.**
(DOCX)
