# Supplementary figures and images for: Basement Membrane-Rich Organoids with Functional Human Blood Vessels Are Permissive Niches for Human Breast Cancer Metastasis
Source: PLoS One. 2013 Aug 8;8(8):e72957. doi: 10.1371/journal.pone.0072957 (PMC3738545; doi:10.1371/journal.pone.0072957)

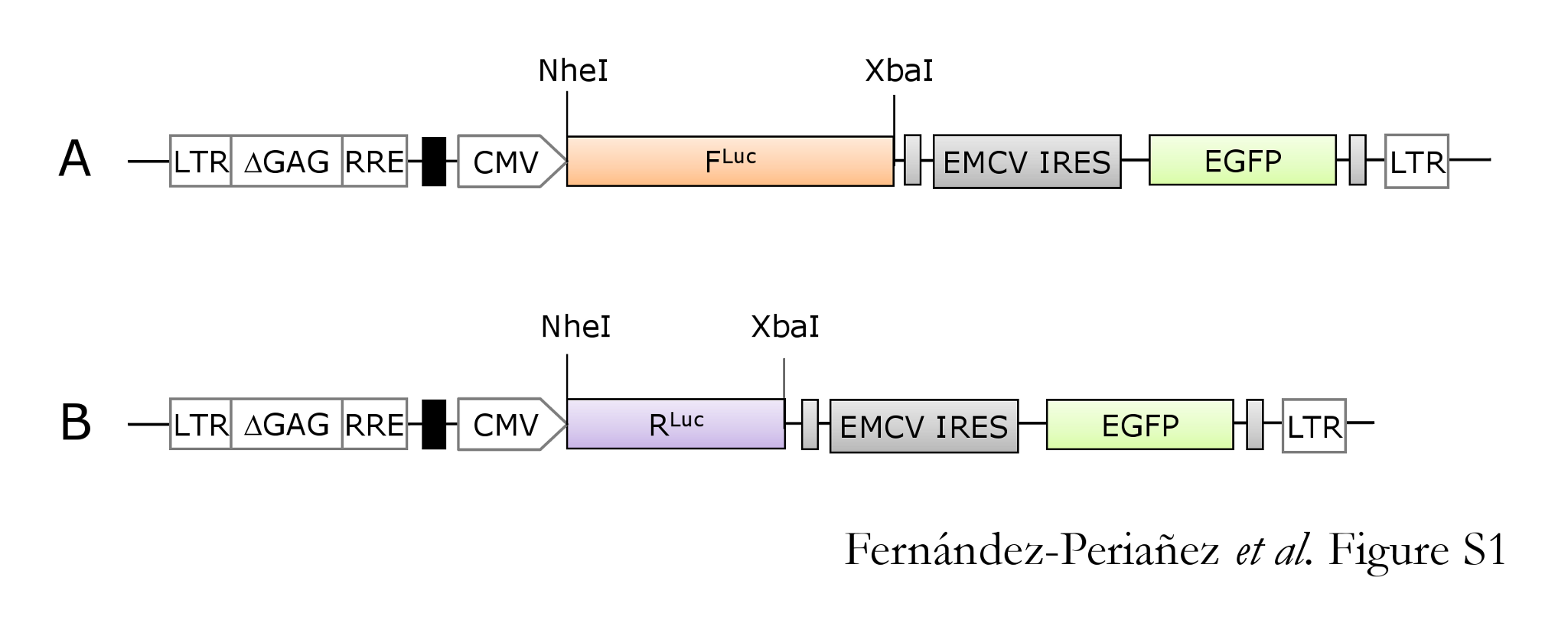

Supplement: Figure S1 — Schematic representation of lentiviral bicistronic vectors. Schematic representation of the lentiviral bicistronic vectors pRRL-FLuc-IRES-EGFP containing firefly luciferase (FLuc) and enhanced-green fluorescent protein (EGFP) genes (a), and pRRL-RLuc-IRES-EGFP containing renilla luciferase (RLuc) and EGFP genes (b). LTR, long terminal repeat; ΔGAG, ATG-deleted group-specific antigen; RRE, Rev-responsive element; EMCV IRES, encephalomyocarditis virus internal ribosomal entry site. (TIF) [file pone.0072957.s001.tif]

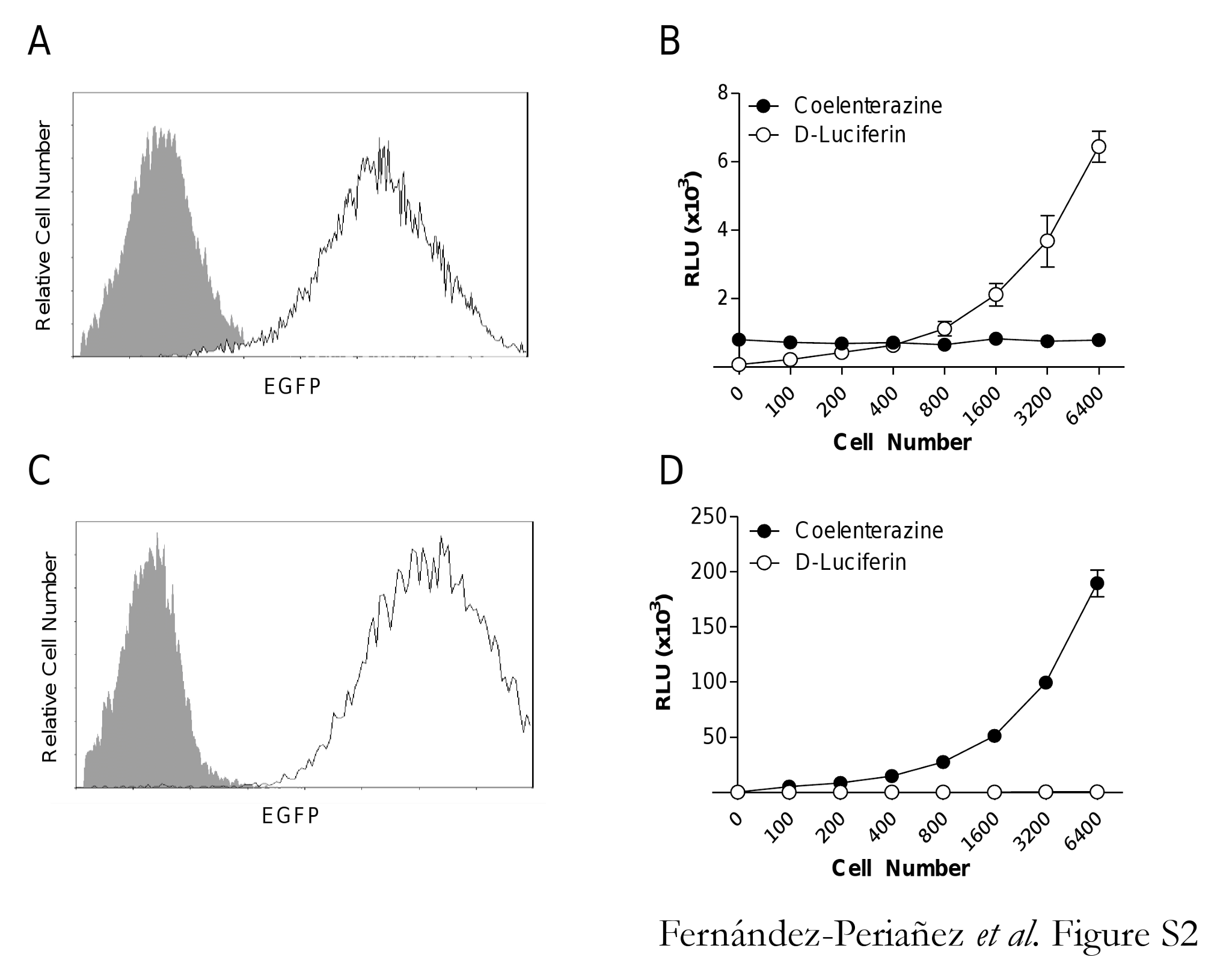

Supplement: Figure S2 — EGFP expression and bioluminescent properties of HUVECFLuc and MDA-MB-231RLuc cells. Flow cytometry analysis showing EGFP expression by lentivirally transduced HUVECFLuc (a) or MDA-MB-231RLuc (c) cells. Bioluminescent properties of HUVECFLuc (b) or MDA-MB-231RLuc cells (d) in the presence and absence of substrate (D-luciferin or coelenterazine). Luciferase activity is expressed as relative light units (RLU). Data represent the average ± SD of triplicate samples. (TIF) [file pone.0072957.s002.tif]

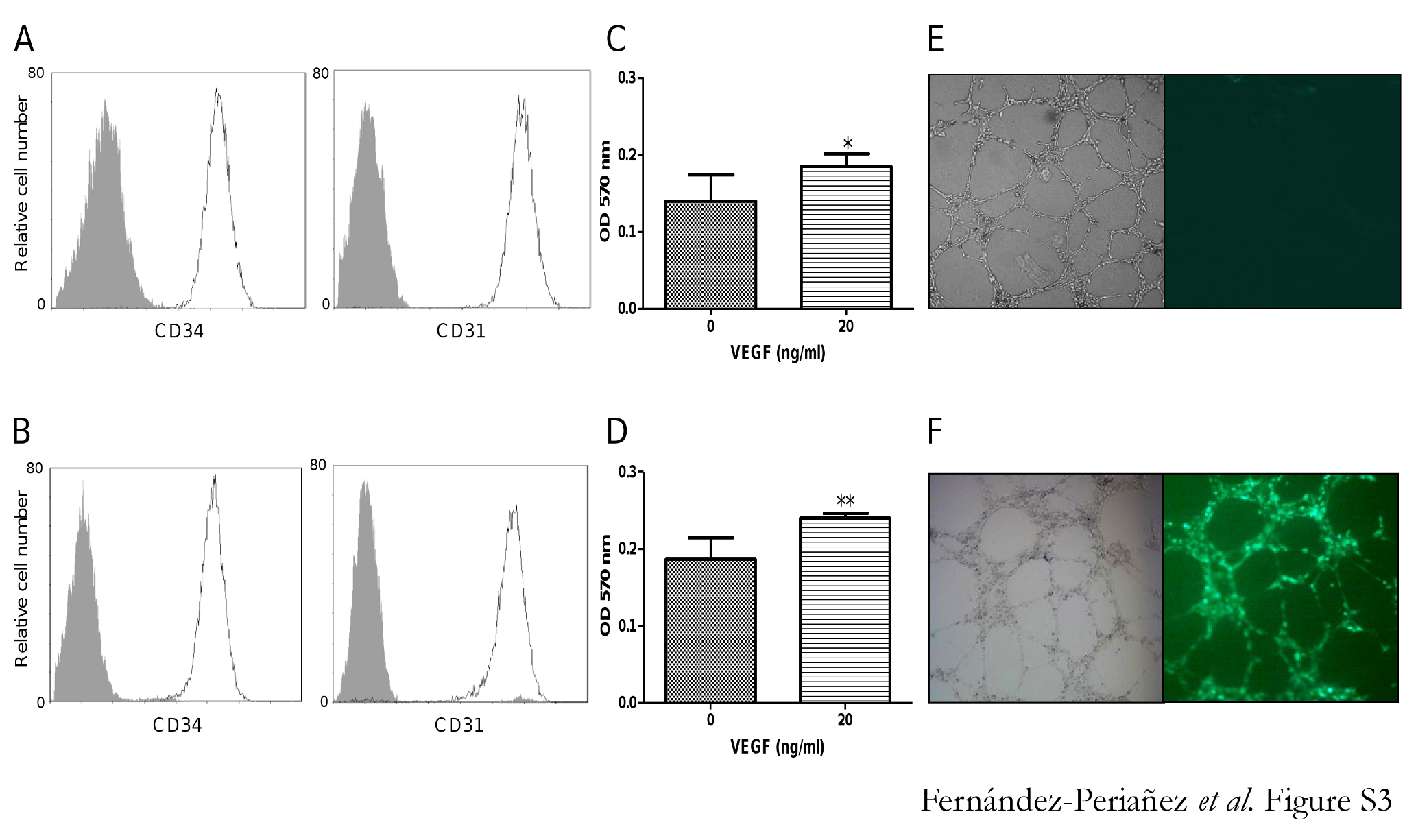

Supplement: Figure S3 — Comparative study of wild-type HUVEC and lentivirally transduced HUVECFLuc. Flow cytometry alaysis of CD34 or CD31 expression (Table S1) on HUVEC (a) and HUVECFLuc (b). Isotype-matched antibodies were used as control (grey line). VEGF significantly induced HUVEC (c) and HUVECFLuc (d) proliferation. Data represent the mean ± SD of triplicate samples. The differences were statistically significant (*p < 0.05, **p < 0.01). Formation of capillary-like structures by HUVEC (e) and HUVECFLuc (f) cultured for 14-16 h on reconstituted Matrigel. (TIF) [file pone.0072957.s003.tif]

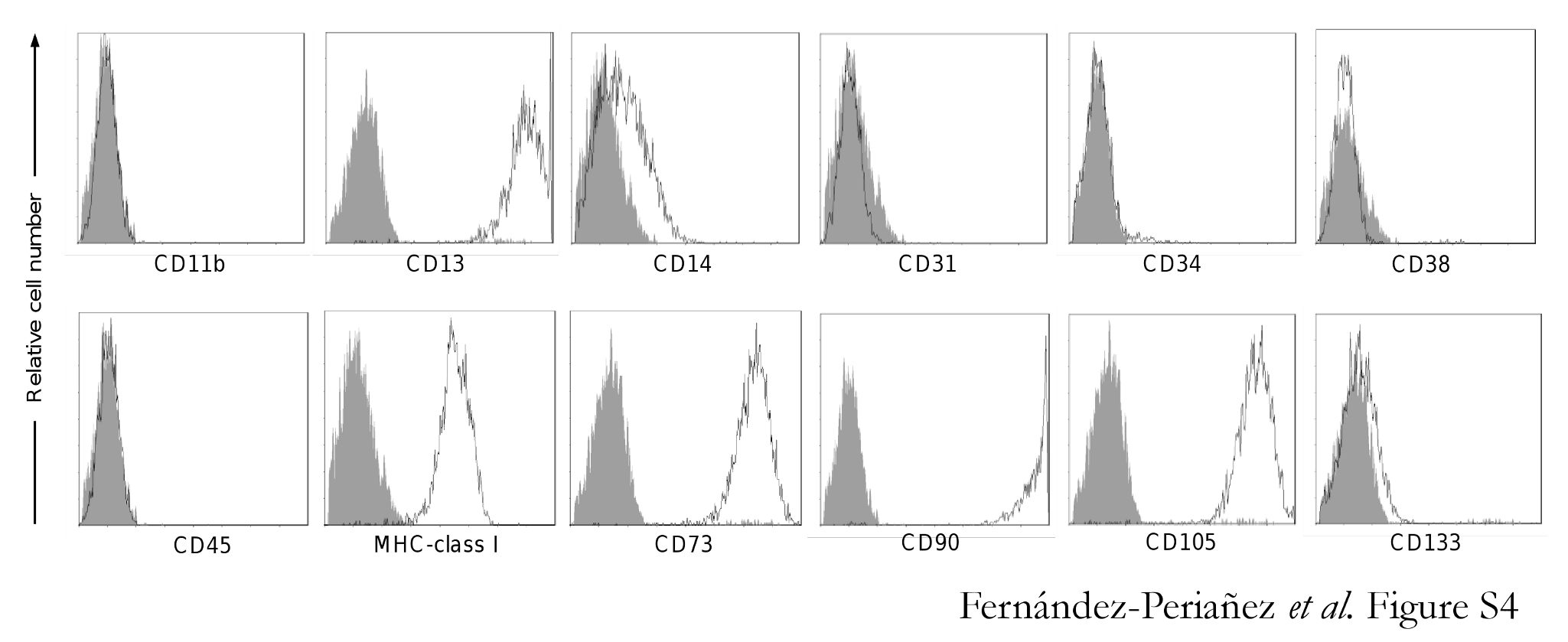

Supplement: Figure S4 — Phenotype of bone marrow-derived human MSC. Cells were trypsinized, labeled with antibodies against the indicated antigens (Table S1) and analyzed by flow cytometry. Isotype-matched antibodies were used as control (grey line). (TIF) [file pone.0072957.s004.tif]

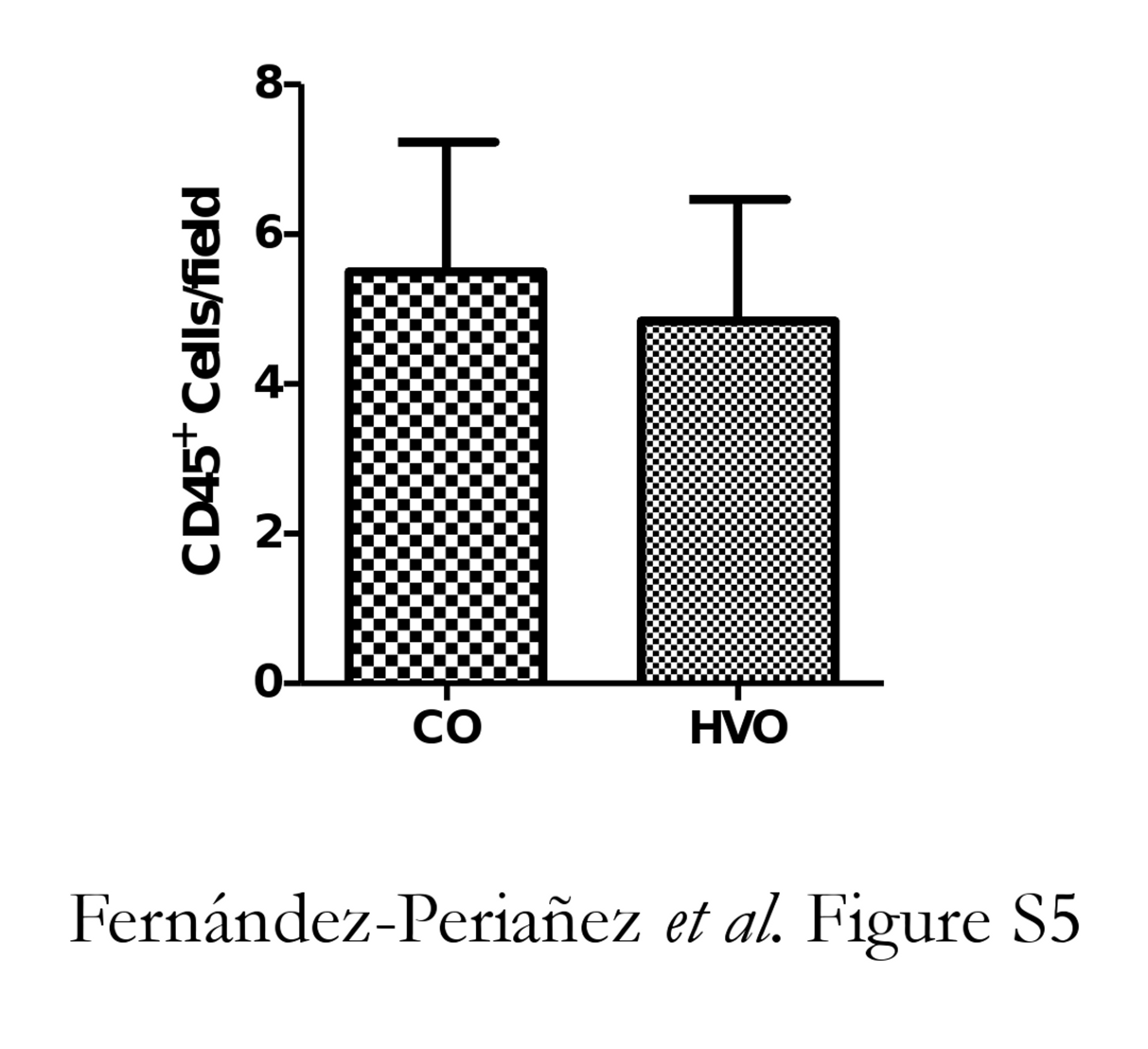

Supplement: Figure S5 — Comparative analysis of CD45+ cells in human vascularized and control BME-rich organoids. Mean ± SD of cells stained with anti-CD45 antibody (Table S3) in four randomly chosen fields (n = 3). (TIF) [file pone.0072957.s005.tif]

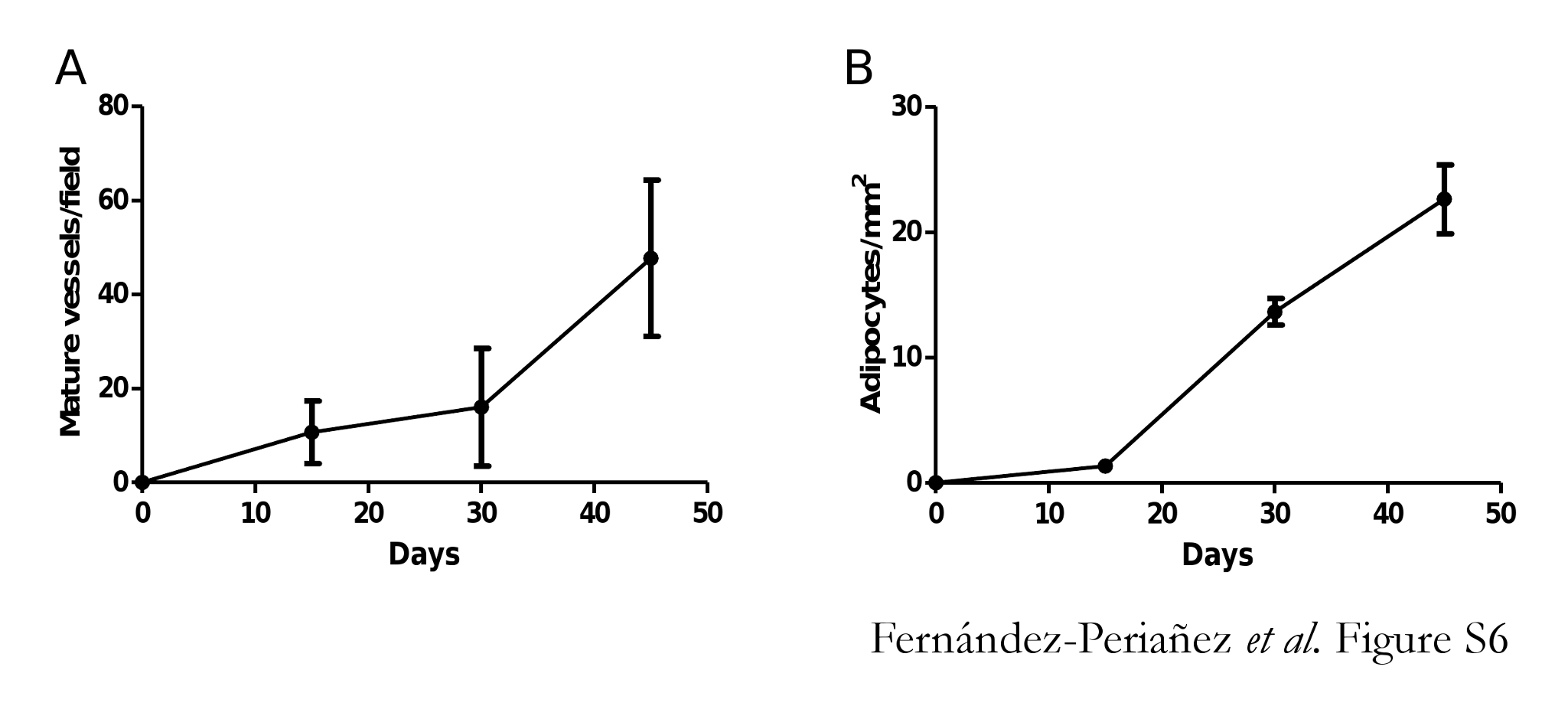

Supplement: Figure S6 — Temporal changes in mature vessel density and in the total adipocyte number in human vascularized BME-rich organoids. Temporal changes in mature vessel density (mean ± SD of perfused vessels in four randomly chosen fields, n = 3) (a), and the number of adipocytes per square millimeter (mean ± SD, n = 3) (b). (TIF) [file pone.0072957.s006.tif]

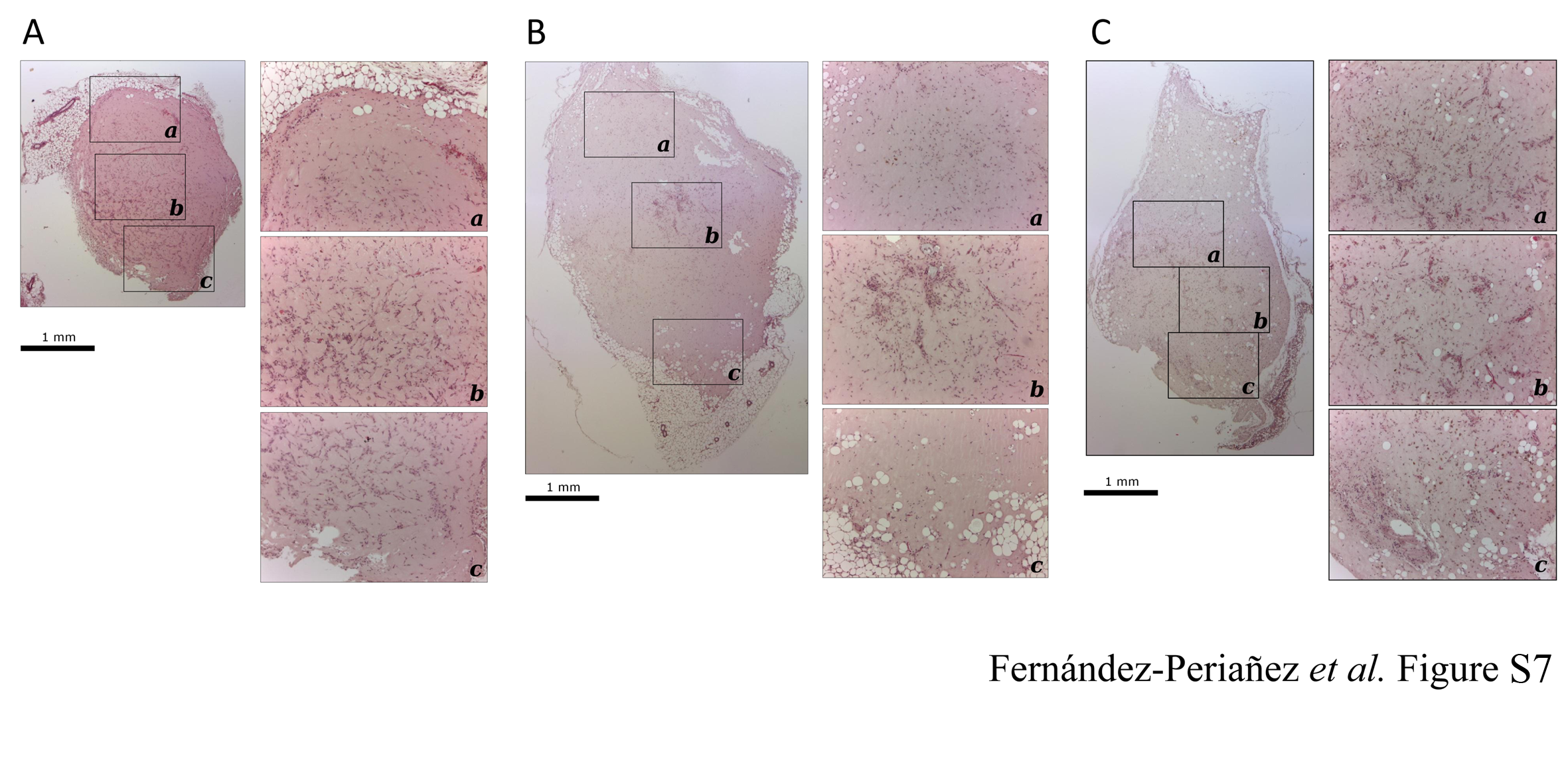

Supplement: Figure S7 — Hematoxylin and eosin-stained sections of human vascularized BME-rich organoids. 15 days (a), 30 days (b), and 45 days after implantation. 4x and 10x images are shown. (TIF) [file pone.0072957.s007.tif]

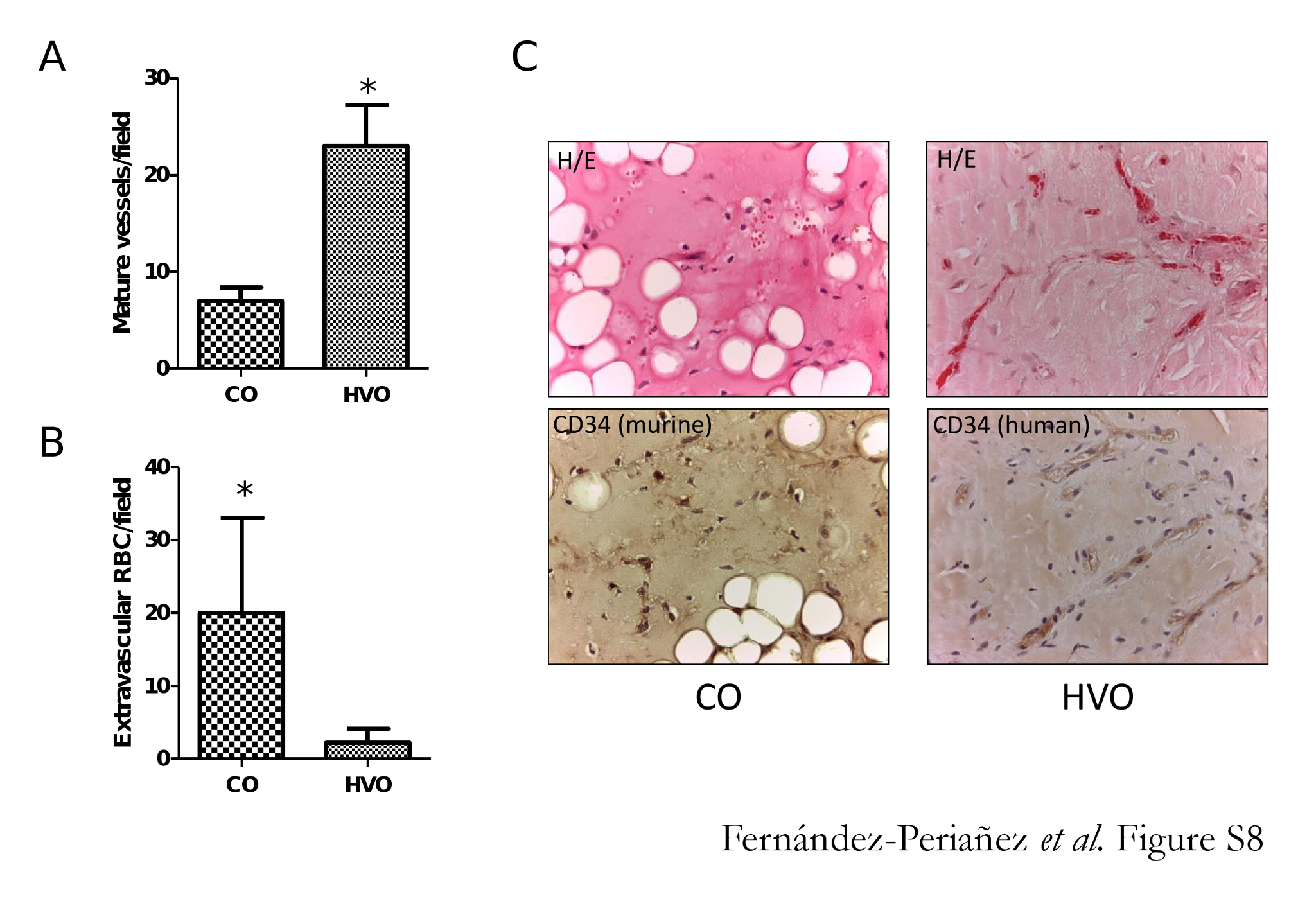

Supplement: Figure S8 — Comparative analysis of mature vessel density and vascular leakiness in human vascularized and control BME-rich organoids 30 days after implantation. (a) Mean ± SD of perfused vessels in four randomly chosen fields (n = 3). (b) Mean ± SD of extravascular red blood cells (RBC) in four randomly chosen fields (n = 3). Significant differences (* p < 0.05). (c) Hematoxylin and eosin-stained sections and immunohystochemical characterization of explanted BME-rich human vascularized and control organoids using anti-CD34 (species specific: human and mouse [39]). (TIF) [file pone.0072957.s008.tif]

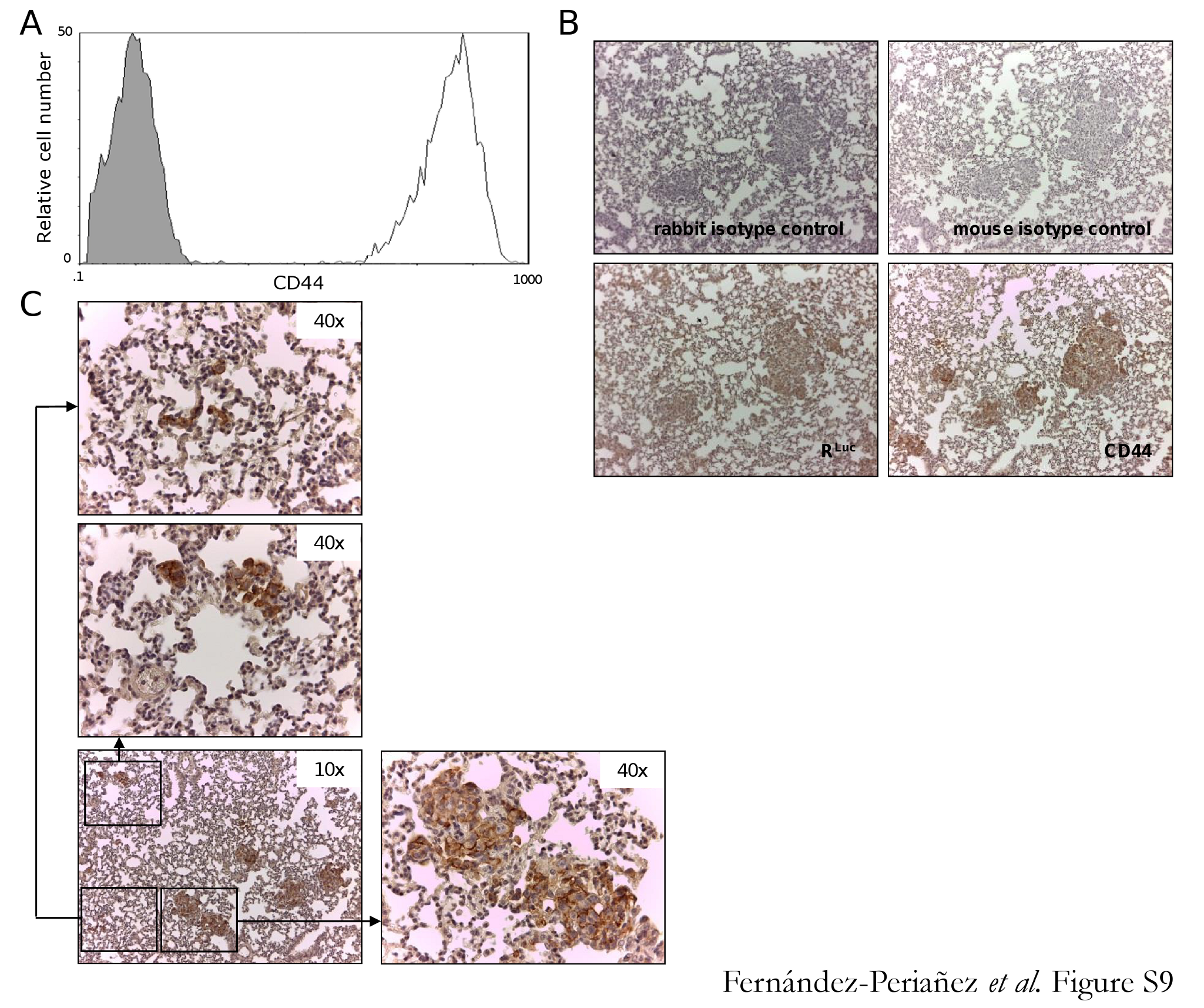

Supplement: Figure S9 — Characterization of antibodies against CD44 and Renilla Luciferase (RLuc). (a) Cell surface expression of CD44 (MEM-85) in MDA-MB-231RLuc cells. (b-c) Immunohistochemical staining of lung metastases. Serial sections of lung tissue were stained for CD44 (F10-44-2) and RLuc. 10x and 40x images are shown. (TIF) [file pone.0072957.s009.tif]
