# Supplementary material for: Basement Membrane-Rich Organoids with Functional Human Blood Vessels Are Permissive Niches for Human Breast Cancer Metastasis
Source: PLoS One. 2013 Aug 8;8(8):e72957. doi: 10.1371/journal.pone.0072957 (PMC3738545; doi:10.1371/journal.pone.0072957)
Supplement: Table S1 — Fluorochrome-conjugated monoclonal antibodies. (DOCX) [file pone.0072957.s010.docx]

| **Clone** | **Specie** | **Target** | **Reactivity** | **Conjugation** | **Supplier** | **Isotype** |
| --- | --- | --- | --- | --- | --- | --- |
| ICRF33 | mouse | CD11b | Human | PE/Cy4 | BD Biosciences^1^ | IgG_1_ |
| WM14 | mouse | CD12 | Human | PE | BD Biosciences^1^ | IgG_1_ |
| UCHM-1 | mouse | CD13 | Human | FITC | Sigma-Aldrich^2^ | IgG_2a_ |
| HEC/64 | mouse | CD21 | Human | FITC | Inmunotools^3^ | IgG_1_ |
| WM-49 | mouse | CD21 | Human | PE | BD Biosciences^1^ | IgG_1_ |
| 481 | mouse | CD23 | Human | PE/Cy4 | Beckman Coulter^4^ | IgG_1_ |
| HIT1 | mouse | CD28 | Human | FITC | Inmunotools^3^ | IgG_1_ |
| HI20 | mouse | CD34 | Human | PE/Cy4 | Inmunotools^3^ | IgG_1_ |
| MEM-85 | mouse | CD44 | Human | PE | Abcam^5^ | IgG_2b_ |
| AD1 | mouse | CD62 | Human | PE | BD Biosciences^1^ | IgG_1_ |
| 4E10 | mouse | CD90 | Human | PE/Cy4 | BD Biosciences^1^ | IgG_1_ |
| SN5 | mouse | CD104 | Human | PE | eBiosciences^6^ | IgG_1_ |
| AC122 | mouse | CD122 | Human | PE | Miltenyi Biotec^7^ | IgG_1_ |
| W5/21 | mouse | MHC-class I | Human | FITC | Sigma-Aldrich^1^ | IgG_2a_ |

**Table S1.** Fluorochrome-conjugated monoclonal antibodies.

^1^Becton Dickinson Biosciences, Bedford, MA, USA. ^2^Sigma-Aldrich, St. Louis, MO, USA. ^3^InmunoTools GMBH, Friesoythe, Germany. ^4^Beckman Coulter, Fullerton, California, USA. ^5^Abcam, Cambridge, UK. ^6^Biosciences, San Diego, CA, USA. **^7^Miltenyi Biotec GmbH,** Bergisch Gladbach, Germany.
