# Supplementary material for: Basement Membrane-Rich Organoids with Functional Human Blood Vessels Are Permissive Niches for Human Breast Cancer Metastasis
Source: PLoS One. 2013 Aug 8;8(8):e72957. doi: 10.1371/journal.pone.0072957 (PMC3738545; doi:10.1371/journal.pone.0072957)
Supplement: Table S2 — Primer pairs used for real-time quantitative RT-PCR. (DOCX) [file pone.0072957.s011.docx]

| **Name** | **Sequence (5´-3´)** | **Product size (bp)** |
| --- | --- | --- |
| R^Luc^-F | ggagaataacttcttcgtggaaac | 75 |
| R^Luc^-R | gctgcaaattcttctggttctaa | 75 |
| hSDHA-F | tgggaacaagagggcatctg | 86 |
| hSDHA-R | ccaccactgcatcaaattcatg | 86 |
| mSDHA-F | gcagtttcgaggcttcttcg | 89 |
| mSDHA-R | aagtgaaagccgcaggtctg | 89 |

**Table S2.** Primer pairs used for real-time quantitative RT-PCR.
