# Supplementary material for: Basement Membrane-Rich Organoids with Functional Human Blood Vessels Are Permissive Niches for Human Breast Cancer Metastasis
Source: PLoS One. 2013 Aug 8;8(8):e72957. doi: 10.1371/journal.pone.0072957 (PMC3738545; doi:10.1371/journal.pone.0072957)
Supplement: Table S3 — Unconjugated monoclonal antibodies. (DOCX) [file pone.0072957.s012.docx]

**Table S3.** Unconjugated monoclonal antibodies.

| **Clone** | **Specie^1^** | **Target** | **Reactivity^1^** | **Supplier** | **Isotype** |
| --- | --- | --- | --- | --- | --- |
| M1/70 | rat | CD11b | Hu, ms, rb | Abcam^2^ | IgG_2b_ |
| PeCa1 | rat | CD19 | Ms | Inmunotools^3^ | IgG_2a_ |
| QBEnd 10 | ms | CD34 | Hu | Dako^4^ | IgG_1_ |
| MEC14.7 | rat | CD34 | Ms | Inmunotools^3^ | IgG_2a_ |
| F10-44-2 | ms | CD44 | Hu | Abcam^2^ | IgG_2a_ |
| EPR1013Y | rb | CD44 | Hu | Millipore^5^ | IgG |
| 30-F11 | rat | CD45 | Ms | BD Biosciences^6^ | IgG_2b_ |
| RNL-1 | ms | CD56 | Hu, ms, rat | Abcam^2^ | IgG_1_ |
| CI:A3-1 | rat | F4/80 | Ms | Abcam^2^ | IgG2b |
| ER-TR7 | rat | Fibroblasts | Hu, ms | Thermo^7^ | IgG2a |
| NIMP-R14 | rat | Neutrophils | Ms | Abcam^2^ | IgG2b |
| 1A4 | ms | αSMA | Hu, ms, rat | Dako^4^ | IgG2a |
| mAb21 | ms | Firefly luciferase | P. pyralis | Millipore^5^ | IgG1 |
| V9 | ms | Vimentin | Hu | Dako^4^ | IgG1 |

^1^hu: human; ms: mouse; rb: rabbit; P. pyralis: Photinus pyralis firefly luciferase. ^2^Abcam, Cambridge, UK. ^3^InmunoTools GMBH, Friesoythe, Germany. ^4^Dako, Carpinteria, CA, USA. ^5^Millipore, Billerica, MA, USA. ^6^Becton Dickinson Biosciences, Bedford, MA, USA. ^7^Thermo Scientific Pierce, Rockford, IL, USA.
